# Supplementary material for: Hot spot prediction in protein-protein interactions by an ensemble system
Source: BMC Syst Biol. 2018 Dec 31;12(Suppl 9):132. doi: 10.1186/s12918-018-0665-8 (PMC6311905; doi:10.1186/s12918-018-0665-8)
Supplement: Supplementary file 2 — All relevant MATLAB code. A simple Matlab implement of our predictor. (ZIP 19,742 kb) [file 12918_2018_665_MOESM2_ESM.zip › Additional file 2/Read ASA/ASA website/dbMPIKT/4GNKA.html]

 NetSurfP - Protein Surface Accessibility and Secondary Structure Predictions

  

|  |  |  |
| --- | --- | --- |
|  |  | NetSurfP - Protein Surface Accessibility and Secondary Structure Predictions  Technical University of Denmark |

  


---

```
# For publication of results, please cite:
# A generic method for assignment of reliability scores applied to solvent accessibility predictions.
# Bent Petersen, Thomas Nordahl Petersen, Pernille Andersen, Morten Nielsen and Claus Lundegaard
# BMC Structural Biology 2009, 9:51 doi:10.1186/1472-6807-9-51
#
# Column 1: Class assignment - B for buried or E for Exposed - Threshold: 25% exposure, but not based on RSA
# Column 2: Amino acid
# Column 3: Sequence name
# Column 4: Amino acid number
# Column 5: Relative Surface Accessibility - RSA
# Column 6: Absolute Surface Accessibility
# Column 7: Z-fit score for RSA prediction
# Column 8: Probability for Alpha-Helix
# Column 9: Probability for Beta-strand
# Column 10: Probability for Coil
E M  4GNKA                  1    0.433  86.663  -2.226   0.003   0.003   0.994
B A  4GNKA                  2    0.216  23.858  -4.460   0.019   0.141   0.840
B C  4GNKA                  3    0.162  22.787  -3.056   0.019   0.141   0.840
B C  4GNKA                  4    0.229  32.166  -1.890   0.018   0.088   0.893
B L  4GNKA                  5    0.317  57.951  -1.938   0.018   0.019   0.964
B S  4GNKA                  6    0.223  26.136  -0.509   0.005   0.015   0.979
E E  4GNKA                  7    0.595 103.999   0.564   0.246   0.004   0.750
E E  4GNKA                  8    0.609 106.375  -0.401   0.502   0.002   0.495
E A  4GNKA                  9    0.443  48.830  -0.262   0.600   0.003   0.397
E K  4GNKA                 10    0.576 118.463   0.105   0.782   0.003   0.216
E E  4GNKA                 11    0.577 100.732   0.229   0.858   0.002   0.139
E A  4GNKA                 12    0.350  38.614  -0.579   0.858   0.002   0.139
E R  4GNKA                 13    0.487 111.477   1.042   0.923   0.002   0.076
E R  4GNKA                 14    0.627 143.537   1.294   0.923   0.002   0.076
E I  4GNKA                 15    0.322  59.533   0.744   0.923   0.002   0.076
B N  4GNKA                 16    0.161  23.600  -0.173   0.782   0.003   0.216
E D  4GNKA                 17    0.549  79.111   1.510   0.858   0.002   0.139
E E  4GNKA                 18    0.579 101.151   1.698   0.923   0.002   0.076
B I  4GNKA                 19    0.071  13.061   0.317   0.923   0.002   0.076
B E  4GNKA                 20    0.219  38.294   0.127   0.923   0.002   0.076
E R  4GNKA                 21    0.562 128.812   1.374   0.970   0.001   0.030
E Q  4GNKA                 22    0.386  68.922   1.052   0.970   0.001   0.030
B L  4GNKA                 23    0.053   9.741   0.860   0.923   0.002   0.076
E R  4GNKA                 24    0.494 113.034   1.370   0.970   0.001   0.030
E R  4GNKA                 25    0.599 137.102   1.398   0.970   0.001   0.030
E D  4GNKA                 26    0.308  44.455   0.225   0.923   0.002   0.076
E K  4GNKA                 27    0.339  69.712  -0.036   0.923   0.002   0.076
E R  4GNKA                 28    0.573 131.125   0.529   0.938   0.007   0.055
E D  4GNKA                 29    0.600  86.402   0.635   0.879   0.010   0.111
B A  4GNKA                 30    0.269  29.633  -0.395   0.717   0.014   0.269
E R  4GNKA                 31    0.495 113.401   0.066   0.522   0.016   0.462
E R  4GNKA                 32    0.546 125.011   0.363   0.339   0.016   0.645
E E  4GNKA                 33    0.385  67.172   1.082   0.199   0.152   0.649
B L  4GNKA                 34    0.095  17.394   0.854   0.074   0.484   0.442
B K  4GNKA                 35    0.209  42.888   1.331   0.018   0.846   0.136
B L  4GNKA                 36    0.023   4.266   0.312   0.006   0.962   0.032
B L  4GNKA                 37    0.048   8.825   0.470   0.006   0.962   0.032
B L  4GNKA                 38    0.025   4.486   0.325   0.011   0.918   0.071
B L  4GNKA                 39    0.044   8.038  -0.024   0.002   0.816   0.182
B G  4GNKA                 40    0.032   2.487  -1.342   0.004   0.514   0.481
B T  4GNKA                 41    0.143  19.820  -0.300   0.004   0.085   0.910
E G  4GNKA                 42    0.296  23.335  -1.016   0.018   0.019   0.964
E E  4GNKA                 43    0.423  73.951  -1.346   0.058   0.017   0.925
B S  4GNKA                 44    0.107  12.587  -1.904   0.115   0.016   0.868
B G  4GNKA                 45    0.086   6.800  -2.224   0.181   0.016   0.803
B K  4GNKA                 46    0.202  41.551  -0.575   0.430   0.016   0.555
B S  4GNKA                 47    0.163  19.104  -1.095   0.660   0.049   0.291
B T  4GNKA                 48    0.147  20.389   0.375   0.879   0.010   0.111
B F  4GNKA                 49    0.073  14.611  -0.068   0.938   0.007   0.055
B I  4GNKA                 50    0.073  13.486   0.421   0.938   0.007   0.055
E K  4GNKA                 51    0.283  58.254   0.332   0.938   0.007   0.055
B Q  4GNKA                 52    0.200  35.720  -0.108   0.938   0.007   0.055
B M  4GNKA                 53    0.046   9.285   0.163   0.879   0.010   0.111
E R  4GNKA                 54    0.315  72.043   0.909   0.938   0.007   0.055
E I  4GNKA                 55    0.339  62.752   0.074   0.879   0.010   0.111
E I  4GNKA                 56    0.230  42.606  -0.234   0.694   0.003   0.303
B H  4GNKA                 57    0.258  46.948   0.068   0.246   0.004   0.750
E G  4GNKA                 58    0.559  44.025  -0.148   0.016   0.005   0.979
E S  4GNKA                 59    0.715  83.857  -0.772   0.016   0.005   0.979
E G  4GNKA                 60    0.586  46.102  -1.131   0.016   0.005   0.979
B Y  4GNKA                 61    0.136  29.170  -0.684   0.016   0.005   0.979
E S  4GNKA                 62    0.399  46.821   0.995   0.053   0.005   0.942
E D  4GNKA                 63    0.589  84.860   0.605   0.923   0.002   0.076
E E  4GNKA                 64    0.658 114.865   0.456   0.923   0.002   0.076
B D  4GNKA                 65    0.186  26.788  -0.002   0.923   0.002   0.076
B K  4GNKA                 66    0.130  26.782   0.408   0.923   0.002   0.076
E R  4GNKA                 67    0.450 102.981   0.691   0.858   0.002   0.139
E G  4GNKA                 68    0.488  38.382   0.497   0.858   0.002   0.139
B F  4GNKA                 69    0.081  16.277   0.825   0.858   0.002   0.139
B T  4GNKA                 70    0.172  23.843   0.732   0.923   0.002   0.076
E K  4GNKA                 71    0.389  80.017   0.370   0.923   0.002   0.076
B L  4GNKA                 72    0.114  20.928   1.005   0.970   0.001   0.030
B V  4GNKA                 73    0.019   2.874   1.379   0.975   0.003   0.022
B Y  4GNKA                 74    0.114  24.276   0.280   0.970   0.001   0.030
E Q  4GNKA                 75    0.296  52.866   1.139   0.970   0.001   0.030
B N  4GNKA                 76    0.060   8.740   0.337   0.970   0.001   0.030
B I  4GNKA                 77    0.022   3.977   1.002   0.988   0.000   0.012
B F  4GNKA                 78    0.108  21.595   0.202   0.988   0.000   0.012
E T  4GNKA                 79    0.331  45.854   1.149   0.988   0.000   0.012
B A  4GNKA                 80    0.036   4.011  -1.390   0.988   0.000   0.012
B M  4GNKA                 81    0.025   5.063  -0.005   0.988   0.000   0.012
E Q  4GNKA                 82    0.286  51.133   0.636   0.970   0.001   0.030
E A  4GNKA                 83    0.232  25.522   0.768   0.970   0.001   0.030
B M  4GNKA                 84    0.026   5.143   0.633   0.970   0.001   0.030
B I  4GNKA                 85    0.068  12.617  -0.244   0.970   0.001   0.030
E R  4GNKA                 86    0.436  99.752   0.795   0.970   0.001   0.030
B A  4GNKA                 87    0.103  11.340  -1.045   0.970   0.001   0.030
B M  4GNKA                 88    0.066  13.107  -1.335   0.923   0.002   0.076
E D  4GNKA                 89    0.538  77.454  -0.171   0.858   0.002   0.139
E T  4GNKA                 90    0.585  81.084   0.414   0.694   0.003   0.303
B L  4GNKA                 91    0.198  36.309  -0.250   0.339   0.016   0.645
E K  4GNKA                 92    0.549 113.012  -0.401   0.053   0.043   0.903
B I  4GNKA                 93    0.134  24.827  -0.023   0.018   0.088   0.893
E P  4GNKA                 94    0.498  70.609   0.172   0.020   0.205   0.775
B Y  4GNKA                 95    0.151  32.183  -0.377   0.020   0.205   0.775
E K  4GNKA                 96    0.583 119.903  -0.226   0.019   0.141   0.840
E Y  4GNKA                 97    0.452  96.678  -1.509   0.053   0.043   0.903
E E  4GNKA                 98    0.502  87.682  -1.170   0.115   0.016   0.868
E H  4GNKA                 99    0.573 104.211  -1.590   0.109   0.005   0.886
E N  4GNKA                100    0.441  64.592  -0.574   0.406   0.004   0.590
E K  4GNKA                101    0.591 121.548  -0.505   0.782   0.003   0.216
E A  4GNKA                102    0.514  56.610  -0.410   0.802   0.014   0.185
B H  4GNKA                103    0.209  37.944   0.334   0.802   0.014   0.185
B A  4GNKA                104    0.136  15.009  -1.166   0.802   0.014   0.185
E Q  4GNKA                105    0.551  98.391   0.968   0.911   0.033   0.057
B L  4GNKA                106    0.188  34.441   1.243   0.831   0.044   0.125
B V  4GNKA                107    0.085  13.111  -0.622   0.831   0.044   0.125
E R  4GNKA                108    0.368  84.249   0.102   0.751   0.050   0.199
E E  4GNKA                109    0.441  77.043  -0.779   0.701   0.107   0.192
B V  4GNKA                110    0.173  26.590  -0.251   0.268   0.043   0.689
E D  4GNKA                111    0.559  80.523  -0.960   0.113   0.043   0.844
E V  4GNKA                112    0.425  65.246  -1.687   0.058   0.017   0.925
E E  4GNKA                113    0.584 102.042  -1.661   0.058   0.017   0.925
E K  4GNKA                114    0.640 131.566  -1.372   0.058   0.017   0.925
E V  4GNKA                115    0.464  71.271  -1.339   0.115   0.016   0.868
E S  4GNKA                116    0.436  51.123  -0.965   0.181   0.016   0.803
E A  4GNKA                117    0.600  66.153  -0.716   0.257   0.016   0.727
B F  4GNKA                118    0.145  29.021   0.029   0.257   0.016   0.727
B E  4GNKA                119    0.213  37.159  -0.506   0.502   0.002   0.495
E N  4GNKA                120    0.524  76.670   0.507   0.923   0.002   0.076
E P  4GNKA                121    0.539  76.484   0.923   0.923   0.002   0.076
B Y  4GNKA                122    0.043   9.104   0.408   0.970   0.001   0.030
B V  4GNKA                123    0.075  11.574   0.135   0.970   0.001   0.030
E D  4GNKA                124    0.577  83.218   1.476   0.970   0.001   0.030
B A  4GNKA                125    0.153  16.839   0.864   0.970   0.001   0.030
B I  4GNKA                126    0.016   2.904   1.126   0.970   0.001   0.030
E K  4GNKA                127    0.353  72.633   1.146   0.970   0.001   0.030
E S  4GNKA                128    0.467  54.732   1.355   0.923   0.002   0.076
B L  4GNKA                129    0.080  14.593   0.857   0.782   0.003   0.216
B W  4GNKA                130    0.099  23.906  -0.013   0.502   0.002   0.495
E N  4GNKA                131    0.601  88.030  -0.391   0.176   0.004   0.820
B D  4GNKA                132    0.186  26.817  -0.724   0.053   0.005   0.942
E P  4GNKA                133    0.661  93.796  -0.166   0.600   0.003   0.397
E G  4GNKA                134    0.340  26.797  -0.542   0.782   0.003   0.216
B I  4GNKA                135    0.049   9.065   0.260   0.938   0.007   0.055
E Q  4GNKA                136    0.318  56.723   1.244   0.938   0.007   0.055
E E  4GNKA                137    0.433  75.558   1.475   0.938   0.007   0.055
B C  4GNKA                138    0.040   5.602  -0.140   0.938   0.007   0.055
B Y  4GNKA                139    0.143  30.645   0.282   0.879   0.010   0.111
E D  4GNKA                140    0.596  85.869   0.592   0.802   0.014   0.185
E R  4GNKA                141    0.377  86.287   0.120   0.339   0.016   0.645
E R  4GNKA                142    0.499 114.225  -0.947   0.053   0.005   0.942
E R  4GNKA                143    0.492 112.599  -0.543   0.053   0.043   0.903
E E  4GNKA                144    0.493  86.127  -0.091   0.056   0.142   0.802
B Y  4GNKA                145    0.078  16.669   0.099   0.019   0.141   0.840
E Q  4GNKA                146    0.404  72.226   0.564   0.020   0.205   0.775
B L  4GNKA                147    0.071  13.073  -0.327   0.056   0.142   0.802
B S  4GNKA                148    0.270  31.632  -1.049   0.053   0.043   0.903
B D  4GNKA                149    0.317  45.694  -1.672   0.115   0.016   0.868
B S  4GNKA                150    0.177  20.803  -1.024   0.339   0.016   0.645
B T  4GNKA                151    0.066   9.099  -0.822   0.522   0.016   0.462
E K  4GNKA                152    0.341  70.082  -0.521   0.717   0.014   0.269
B Y  4GNKA                153    0.150  32.119   0.133   0.717   0.014   0.269
B Y  4GNKA                154    0.075  16.028  -0.265   0.717   0.014   0.269
B L  4GNKA                155    0.073  13.311  -0.054   0.782   0.003   0.216
E N  4GNKA                156    0.462  67.593   0.601   0.694   0.003   0.303
B D  4GNKA                157    0.191  27.581   0.205   0.858   0.002   0.139
B L  4GNKA                158    0.033   6.097  -0.343   0.923   0.002   0.076
E D  4GNKA                159    0.288  41.458   0.124   0.923   0.002   0.076
E R  4GNKA                160    0.389  89.173   0.751   0.923   0.002   0.076
B V  4GNKA                161    0.072  11.113  -0.528   0.938   0.007   0.055
B A  4GNKA                162    0.179  19.770   0.383   0.802   0.014   0.185
E D  4GNKA                163    0.452  65.104   0.743   0.406   0.004   0.590
E P  4GNKA                164    0.447  63.373   0.062   0.181   0.016   0.803
E S  4GNKA                165    0.569  66.675  -0.271   0.113   0.043   0.844
B Y  4GNKA                166    0.117  24.939   0.634   0.118   0.150   0.732
E L  4GNKA                167    0.299  54.820   0.885   0.064   0.216   0.721
B P  4GNKA                168    0.232  32.921   0.121   0.064   0.216   0.721
E T  4GNKA                169    0.302  41.901   0.556   0.052   0.084   0.864
E Q  4GNKA                170    0.627 112.054  -1.015   0.113   0.043   0.844
E Q  4GNKA                171    0.619 110.625  -0.222   0.053   0.043   0.903
B D  4GNKA                172    0.268  38.547  -0.953   0.056   0.142   0.802
B V  4GNKA                173    0.228  34.967   0.090   0.022   0.552   0.426
B L  4GNKA                174    0.204  37.426  -0.514   0.021   0.756   0.223
B R  4GNKA                175    0.325  74.494  -0.548   0.021   0.756   0.223
B V  4GNKA                176    0.096  14.678   0.023   0.021   0.756   0.223
E R  4GNKA                177    0.398  91.211  -0.268   0.023   0.655   0.322
B V  4GNKA                178    0.173  26.590  -0.093   0.004   0.420   0.576
B P  4GNKA                179    0.246  34.950  -0.283   0.004   0.138   0.858
B T  4GNKA                180    0.118  16.394  -1.132   0.005   0.262   0.733
B T  4GNKA                181    0.241  33.441  -0.175   0.005   0.336   0.660
B G  4GNKA                182    0.157  12.387  -1.834   0.004   0.420   0.576
B I  4GNKA                183    0.098  18.204  -0.027   0.004   0.514   0.481
B I  4GNKA                184    0.219  40.552  -0.569   0.003   0.718   0.279
B E  4GNKA                185    0.252  43.955  -0.298   0.001   0.900   0.099
B Y  4GNKA                186    0.271  58.020   0.407   0.001   0.959   0.040
E P  4GNKA                187    0.405  57.441   0.605   0.001   0.959   0.040
B F  4GNKA                188    0.091  18.244   0.793   0.001   0.900   0.099
E D  4GNKA                189    0.544  78.333   0.256   0.003   0.718   0.279
E L  4GNKA                190    0.313  57.310  -0.127   0.005   0.336   0.660
E Q  4GNKA                191    0.728 130.021  -0.494   0.005   0.015   0.979
E S  4GNKA                192    0.691  81.020  -0.382   0.005   0.015   0.979
B V  4GNKA                193    0.165  25.361   0.909   0.004   0.616   0.381
E I  4GNKA                194    0.347  64.158   0.694   0.001   0.900   0.099
B F  4GNKA                195    0.056  11.219   0.532   0.001   0.900   0.099
B R  4GNKA                196    0.160  36.709   0.466   0.011   0.918   0.071
B M  4GNKA                197    0.029   5.783  -0.243   0.018   0.846   0.136
B V  4GNKA                198    0.146  22.456   0.473   0.021   0.756   0.223
B D  4GNKA                199    0.068   9.799  -2.904   0.004   0.616   0.381
B V  4GNKA                200    0.083  12.803  -0.990   0.022   0.359   0.619
B G  4GNKA                201    0.170  13.403  -1.881   0.018   0.088   0.893
B G  4GNKA                202    0.261  20.501  -2.215   0.018   0.019   0.964
E Q  4GNKA                203    0.322  57.491  -0.710   0.018   0.047   0.935
E R  4GNKA                204    0.484 110.790  -0.917   0.053   0.043   0.903
E S  4GNKA                205    0.492  57.627  -1.564   0.053   0.043   0.903
B E  4GNKA                206    0.181  31.533  -1.661   0.113   0.043   0.844
B R  4GNKA                207    0.230  52.647  -0.394   0.184   0.043   0.773
E R  4GNKA                208    0.465 106.554  -1.055   0.268   0.043   0.689
B K  4GNKA                209    0.262  53.852  -0.912   0.354   0.048   0.598
B W  4GNKA                210    0.050  12.025  -0.544   0.455   0.046   0.498
E I  4GNKA                211    0.310  57.276   0.686   0.605   0.105   0.290
E H  4GNKA                212    0.392  71.341   0.072   0.502   0.102   0.396
B C  4GNKA                213    0.089  12.482  -0.446   0.502   0.102   0.396
B F  4GNKA                214    0.052  10.456  -0.273   0.278   0.093   0.628
E E  4GNKA                215    0.583 101.885  -0.761   0.113   0.043   0.844
E N  4GNKA                216    0.477  69.847  -0.808   0.018   0.047   0.935
B V  4GNKA                217    0.109  16.692  -0.254   0.005   0.045   0.951
B T  4GNKA                218    0.207  28.739  -0.099   0.005   0.262   0.733
B S  4GNKA                219    0.023   2.731   0.544   0.002   0.816   0.182
B I  4GNKA                220    0.016   2.923   1.407   0.001   0.959   0.040
B M  4GNKA                221    0.013   2.661   1.652   0.001   0.959   0.040
B F  4GNKA                222    0.021   4.295   1.361   0.001   0.959   0.040
B L  4GNKA                223    0.012   2.270   1.628   0.001   0.900   0.099
B V  4GNKA                224    0.024   3.735   0.676   0.003   0.718   0.279
B A  4GNKA                225    0.055   6.061  -1.937   0.004   0.420   0.576
B L  4GNKA                226    0.038   7.049  -1.547   0.005   0.045   0.951
B S  4GNKA                227    0.104  12.201  -1.042   0.018   0.019   0.964
B E  4GNKA                228    0.239  41.683  -0.933   0.058   0.017   0.925
E Y  4GNKA                229    0.297  63.490  -0.703   0.115   0.016   0.868
E D  4GNKA                230    0.365  52.669  -1.694   0.113   0.043   0.844
B Q  4GNKA                231    0.262  46.829  -0.059   0.125   0.227   0.648
B V  4GNKA                232    0.229  35.167  -1.037   0.135   0.317   0.548
B L  4GNKA                233    0.210  38.396  -0.188   0.231   0.330   0.439
E V  4GNKA                234    0.399  61.403  -0.522   0.352   0.332   0.316
E E  4GNKA                235    0.471  82.214  -1.223   0.321   0.252   0.427
E S  4GNKA                236    0.311  36.437  -0.735   0.191   0.086   0.723
E D  4GNKA                237    0.561  80.912  -0.717   0.184   0.043   0.773
E N  4GNKA                238    0.578  84.590  -0.822   0.268   0.043   0.689
E E  4GNKA                239    0.369  64.447   0.771   0.268   0.043   0.689
B N  4GNKA                240    0.251  36.717  -0.604   0.268   0.043   0.689
B R  4GNKA                241    0.158  36.228   1.300   0.717   0.014   0.269
B M  4GNKA                242    0.049   9.805  -0.020   0.938   0.007   0.055
E E  4GNKA                243    0.433  75.593   0.938   0.970   0.001   0.030
E E  4GNKA                244    0.257  44.985   0.857   0.970   0.001   0.030
B S  4GNKA                245    0.017   1.957   0.720   0.970   0.001   0.030
B K  4GNKA                246    0.174  35.710   1.018   0.970   0.001   0.030
E A  4GNKA                247    0.551  60.720   1.476   0.970   0.001   0.030
B L  4GNKA                248    0.115  21.148   1.216   0.988   0.000   0.012
B F  4GNKA                249    0.020   4.114   0.925   0.988   0.000   0.012
E R  4GNKA                250    0.364  83.379   1.962   0.970   0.001   0.030
E T  4GNKA                251    0.416  57.741   1.716   0.970   0.001   0.030
B I  4GNKA                252    0.043   7.900   0.622   0.923   0.002   0.076
B I  4GNKA                253    0.064  11.785   0.402   0.782   0.003   0.216
E T  4GNKA                254    0.640  88.810   0.614   0.321   0.003   0.675
E Y  4GNKA                255    0.281  60.071   0.619   0.109   0.005   0.886
E P  4GNKA                256    0.708 100.437   0.635   0.176   0.004   0.820
E W  4GNKA                257    0.329  79.124   0.469   0.257   0.016   0.727
B F  4GNKA                258    0.041   8.209  -0.464   0.257   0.016   0.727
E Q  4GNKA                259    0.404  72.154   1.142   0.181   0.016   0.803
E N  4GNKA                260    0.704 103.080   1.025   0.058   0.017   0.925
B S  4GNKA                261    0.035   4.149   1.188   0.018   0.088   0.893
B S  4GNKA                262    0.067   7.829   0.628   0.021   0.451   0.528
B V  4GNKA                263    0.023   3.566   1.178   0.018   0.846   0.136
B I  4GNKA                264    0.019   3.496   1.632   0.011   0.918   0.071
B L  4GNKA                265    0.018   3.332   1.534   0.011   0.918   0.071
B F  4GNKA                266    0.025   5.058   0.998   0.018   0.846   0.136
B L  4GNKA                267    0.019   3.552   0.805   0.023   0.655   0.322
B N  4GNKA                268    0.097  14.142   0.423   0.069   0.386   0.545
B K  4GNKA                269    0.170  35.072   0.715   0.278   0.093   0.628
E K  4GNKA                270    0.252  51.754   0.985   0.354   0.048   0.598
E D  4GNKA                271    0.261  37.653  -0.495   0.522   0.016   0.462
B L  4GNKA                272    0.153  28.106   0.367   0.717   0.014   0.269
B L  4GNKA                273    0.095  17.449   0.230   0.802   0.014   0.185
E E  4GNKA                274    0.490  85.586   1.365   0.802   0.014   0.185
E E  4GNKA                275    0.618 108.052   0.571   0.717   0.014   0.269
B K  4GNKA                276    0.240  49.389   0.316   0.622   0.015   0.363
B I  4GNKA                277    0.086  15.928  -0.715   0.430   0.016   0.555
E M  4GNKA                278    0.563 112.716   0.268   0.181   0.016   0.803
E Y  4GNKA                279    0.588 125.570  -0.133   0.113   0.043   0.844
B S  4GNKA                280    0.275  32.195  -0.122   0.053   0.043   0.903
E H  4GNKA                281    0.550 100.118   0.631   0.018   0.047   0.935
B L  4GNKA                282    0.073  13.366  -1.067   0.052   0.084   0.864
E V  4GNKA                283    0.441  67.828   0.668   0.056   0.142   0.802
E D  4GNKA                284    0.625  90.062   0.610   0.064   0.216   0.721
B Y  4GNKA                285    0.174  37.077   0.646   0.056   0.142   0.802
B F  4GNKA                286    0.076  15.313   0.362   0.018   0.088   0.893
E P  4GNKA                287    0.472  66.977  -0.421   0.018   0.047   0.935
E E  4GNKA                288    0.619 108.104  -0.436   0.018   0.047   0.935
B Y  4GNKA                289    0.144  30.794  -0.541   0.018   0.047   0.935
E D  4GNKA                290    0.658  94.775  -0.762   0.018   0.019   0.964
E G  4GNKA                291    0.552  43.474  -2.065   0.003   0.003   0.994
E P  4GNKA                292    0.528  74.895  -1.066   0.016   0.005   0.979
E Q  4GNKA                293    0.467  83.406  -1.358   0.053   0.005   0.942
E R  4GNKA                294    0.437  99.981  -1.129   0.058   0.017   0.925
B D  4GNKA                295    0.226  32.567  -0.524   0.321   0.003   0.675
B A  4GNKA                296    0.108  11.858  -0.550   0.923   0.002   0.076
E Q  4GNKA                297    0.552  98.587   0.402   0.970   0.001   0.030
E A  4GNKA                298    0.350  38.548   1.154   0.970   0.001   0.030
B A  4GNKA                299    0.019   2.083   0.435   0.970   0.001   0.030
B R  4GNKA                300    0.124  28.465   0.915   0.970   0.001   0.030
E E  4GNKA                301    0.482  84.205   1.583   0.970   0.001   0.030
B F  4GNKA                302    0.142  28.519   1.287   0.988   0.000   0.012
B I  4GNKA                303    0.031   5.717   0.538   0.988   0.000   0.012
B L  4GNKA                304    0.199  36.364   1.491   0.988   0.000   0.012
E K  4GNKA                305    0.493 101.390   1.188   0.988   0.000   0.012
B M  4GNKA                306    0.241  48.324   1.825   0.970   0.001   0.030
B F  4GNKA                307    0.050  10.035   0.447   0.970   0.001   0.030
E V  4GNKA                308    0.370  56.915   1.345   0.923   0.002   0.076
E D  4GNKA                309    0.635  91.475   0.489   0.858   0.002   0.139
B L  4GNKA                310    0.219  40.062  -0.244   0.694   0.003   0.303
B N  4GNKA                311    0.351  51.342  -0.386   0.406   0.004   0.590
E P  4GNKA                312    0.635  90.121   0.216   0.181   0.016   0.803
E D  4GNKA                313    0.537  77.439  -0.248   0.058   0.017   0.925
E S  4GNKA                314    0.630  73.848  -0.352   0.018   0.019   0.964
E D  4GNKA                315    0.779 112.254  -0.015   0.005   0.015   0.979
B K  4GNKA                316    0.216  44.472   0.519   0.004   0.085   0.910
E I  4GNKA                317    0.375  69.431   1.508   0.004   0.616   0.381
B I  4GNKA                318    0.028   5.198   0.892   0.001   0.900   0.099
B Y  4GNKA                319    0.068  14.553   0.726   0.001   0.959   0.040
B S  4GNKA                320    0.039   4.606   0.149   0.001   0.900   0.099
B H  4GNKA                321    0.068  12.333   0.716   0.001   0.900   0.099
B F  4GNKA                322    0.070  14.149   0.108   0.018   0.846   0.136
B T  4GNKA                323    0.040   5.520  -0.130   0.021   0.756   0.223
B C  4GNKA                324    0.064   8.986  -0.001   0.021   0.756   0.223
B A  4GNKA                325    0.032   3.537  -0.893   0.023   0.655   0.322
B T  4GNKA                326    0.129  17.962   0.340   0.022   0.359   0.619
B D  4GNKA                327    0.181  26.053  -0.110   0.056   0.142   0.802
E T  4GNKA                328    0.228  31.637  -0.277   0.600   0.003   0.397
E E  4GNKA                329    0.567  99.090   0.459   0.694   0.003   0.303
B N  4GNKA                330    0.105  15.343   0.207   0.782   0.003   0.216
B I  4GNKA                331    0.022   4.089   1.408   0.970   0.001   0.030
E R  4GNKA                332    0.342  78.410   1.650   0.970   0.001   0.030
E F  4GNKA                333    0.330  66.331   1.712   0.988   0.000   0.012
B V  4GNKA                334    0.026   4.042   0.846   0.988   0.000   0.012
B F  4GNKA                335    0.035   7.105   0.671   0.988   0.000   0.012
E A  4GNKA                336    0.470  51.805   2.050   0.988   0.000   0.012
B A  4GNKA                337    0.202  22.304   1.273   0.988   0.000   0.012
B V  4GNKA                338    0.018   2.736   1.266   0.988   0.000   0.012
B K  4GNKA                339    0.172  35.339   1.238   0.988   0.000   0.012
E D  4GNKA                340    0.382  55.046   1.252   0.988   0.000   0.012
B T  4GNKA                341    0.105  14.550   1.320   0.988   0.000   0.012
B I  4GNKA                342    0.028   5.254   0.710   0.988   0.000   0.012
B L  4GNKA                343    0.122  22.338   0.745   0.970   0.001   0.030
E Q  4GNKA                344    0.427  76.298   1.354   0.970   0.001   0.030
E L  4GNKA                345    0.361  66.044   1.382   0.923   0.002   0.076
B N  4GNKA                346    0.125  18.300   0.689   0.923   0.002   0.076
B L  4GNKA                347    0.132  24.206  -0.132   0.858   0.002   0.139
E K  4GNKA                348    0.562 115.665   0.939   0.858   0.002   0.139
E E  4GNKA                349    0.672 117.416   0.545   0.694   0.003   0.303
B Y  4GNKA                350    0.220  47.014   0.033   0.321   0.003   0.675
E N  4GNKA                351    0.626  91.646  -0.688   0.109   0.005   0.886
B L  4GNKA                352    0.184  33.672  -0.071   0.018   0.019   0.964
E V  4GNKA                353    0.581  89.300  -1.522   0.003   0.003   0.994

Explain the output.  Go back.
```

---
